# Our Name

**Published:** 1861-08

**Authors:** 


### OUR NAME.

Since this journal is ready for the press, we are very politely notified that we must not call it *The Buffalo Medical Journal*, since Mr. A. I. Mathews claims to own that name, and designs to use it himself. We have no desire to use that name, and not the slightest objection to Mr. Mathew's making as free use of it as he pleases; the only suggestion we desire to make, is, that if Mr. Mathews should really publish a journal, the name most appropriate, expressive and highly promotive of his present business interests would certainly seem to be The Buffalo *Patent Medicine* Journal. We shall endeavor to fill the hiatus made by the discontinuance or removal of the former Buffalo Journal, with *legitimate medicine.*

